# Trial protocol to compare the efficacy of a smartphone-based blood glucose management system with standard clinic care in the gestational diabetic population

**DOI:** 10.1136/bmjopen-2015-009702

**Published:** 2016-03-17

**Authors:** Lucy H Mackillop, Katy Bartlett, Jacqueline Birks, Andrew J Farmer, Oliver J Gibson, Dev A Kevat, Yvonne Kenworthy, Jonathan C Levy, Lise Loerup, Lionel Tarassenko, Carmelo Velardo, Jane E Hirst

**Affiliations:** 1 Nuffield Department of Obstetrics & Gynaecology, Level 3, Women's Centre, John Radcliffe Hospital, Oxford, UK; 2 Women's Centre, Oxford University Hospitals NHS Trust, Oxford, UK; 3 Centre for Statistics in Medicine, University of Oxford, Oxford, UK; 4 Nuffield Department of Primary Care Health Sciences, University of Oxford, Oxford, UK; 5 Department of Engineering Science, University of Oxford, Oxford, UK; 6 Department of Endocrinology, Royal Brisbane and Women's Hospital, Herston, Queensland, Australia; 7 School of Public Health, Monash University, Melbourne, Australia; 8 Nuffield Department of Obstetrics & Gynaecology, Oxford University Hospitals NHS Trust, Oxford, UK; 9 The Oxford Centre for Diabetes, Endocrinology and Metabolism, Oxford University Hospitals NHS Trust, Oxford, UK

## Abstract

**Introduction:**

The prevalence of gestational diabetes mellitus (GDM) is rising in the UK. Good glycaemic control improves maternal and neonatal outcomes. Frequent clinical review of patients with GDM by healthcare professionals is required owing to the rapidly changing physiology of pregnancy and its unpredictable course. Novel technologies that allow home blood glucose (BG) monitoring with results transmitted in real time to a healthcare professional have the potential to deliver good-quality healthcare to women more conveniently and at a lower cost to the patient and the healthcare provider compared to the conventional face-to-face or telephone-based consultation. We have developed an integrated GDm-health management system and aim to test the impact of using this system on maternal glycaemic control, costs, patient satisfaction and maternal and neonatal outcomes compared to standard clinic care in a single large publicly funded (National Health Service (NHS)) maternity unit.

**Methods and analysis:**

Women with confirmed gestational diabetes in a current pregnancy are individually randomised to either the GDm-health system and half the normal clinic visits or normal clinic care. Primary outcome is mean BG in each group from recruitment to delivery calculated, with adjustments made for number of BG measurements, proportion of preprandial and postprandial readings and length of time in study, and compared between the groups. The secondary objective will be to compare the two groups for compliance to the allocated BG monitoring regime, maternal and neonatal outcomes, glycaemic control using glycated haemoglobin (HbA1c) and other BG metrics, and patient attitudes to care assessed using a questionnaire and resource use.

**Ethics and dissemination:**

Thresholds for treatment, dietary advice and clinical management are the same in both groups. The results of the study will be published in a peer-reviewed journal and disseminated electronically and in print.

**Trial registration number:**

NCT01916694; Pre-results.

**Strengths and limitations of this study**

- This will be the most rigorous and robust evaluation of an integrated telehealth solution for the gestational diabetes population to date.
- The intervention has been developed and piloted prior to the study with input from consumer groups: patients, clinicians and biomedical engineers.
- This study will be conducted within the maternity diabetes service of a National Health Service (NHS) hospital, therefore capturing data in a ‘real-life’ scenario.
- As adverse clinical outcomes such as shoulder dystocia, birth trauma or stillbirth are relatively uncommon in women with gestational diabetes mellitus receiving treatment, this study will not be able to be powered on these outcomes.
- The optimal metric for measuring glycaemic control in the gestational diabetic population is not known. We plan to assess the difference in overall mean blood glucose between the two groups as the primary outcome for this trial; however, an intended objective will also be to assess and compare other summative metrics of glucose control. This will enable us to select a valid primary outcome measure, with known SD and estimation of effect size, to allow calculation of sample size for a future definitive trial.
- The study will be conducted in a single UK tertiary centre where women have high rates of literacy and relatively low levels of social deprivation; therefore, further evaluation will be needed to demonstrate effectiveness in other settings.

## Introduction

Gestational diabetes mellitus (GDM) is defined by the WHO as ‘carbohydrate intolerance resulting in hyperglycaemia of variable severity with onset or first recognition during pregnancy’.^1^ Maternal hyperglycaemia leads to excess transfer of glucose to the fetus resulting in fetal hyperinsulinaemia. The consequence of this is accelerated fetal growth and large babies, which increases the risk of delivery complications such as shoulder dystocia, birth trauma, the need for caesarean section and increased risk of stillbirth.^2^ Recognising and treating gestational diabetes to achieve tight glycaemic control has been shown in randomised controlled trials to reduce obstetric and fetal complications.^3^ ^4^

Blood glucose (BG) metabolism and regulation change rapidly in pregnancy. The development of GDM and its progression to requiring pharmacologic treatment can be difficult to predict accurately. Once a woman is on insulin treatment, rapid upward titration of insulin dose is commonly required to maintain optimal glycaemic control and rarely hypoglycaemic episodes may require reduction in insulin dosages.

In order to detect and respond to these physiological and metabolic changes, women with GDM are commonly reviewed by a hospital-based maternity diabetes team, at frequent and regular intervals—typically every 1–4 weeks. This constitutes a significant burden to the patient of having to attend many additional antenatal clinics and to the health system responsible for care.

In recent years, there has been a widening of GDM screening criteria,^5^ a lowering of diagnostic thresholds,^6^ an increasing proportion of pregnant women who are classed overweight or obese and increasing numbers of women from high-risk ethnic groups. These changes have led to a rise in the prevalence of GDM, from a baseline of around 4% in 2008^5^ predicted to reach over 16% in the UK if the new diagnostic thresholds are widely adopted.^7^ Taking these factors together, current maternity diabetes services are likely to become overwhelmed and provision and funding will be required for rapid expansion of these services.

The concept of women being able to monitor their BG levels at home with results transmitted in real time to a healthcare provider with bidirectional communication is attractive. Technology intended to deliver healthcare at a distance using an electronic means of communication is referred to as digital health. M-health is a subcategory of digital health where smartphones are used to transfer data and run applications to integrate with other health technology and services.

We developed a novel m-health management system for women with GDM (GDm-health). This system was designed to be an intuitive, interactive, reliable and accurate digital health solution for women with GDM.^8^ GDm-health was designed to include a smartphone application for the patients and a web interface for the clinicians. It incorporates several features of digital health technology that have only been evaluated in isolation before, including bidirectional communication, integration with an existing health system and digital recording and analysis of BG results. The system was developed in collaboration with women with GDM, healthcare professionals and biomedical engineers to create a system that could potentially be fully integrated into routine clinical care within the National Health Service (NHS), with an interface that was easy to use for staff and patients. Full details of development of the system are provided elsewhere.^8^ Additionally, the system needed to be fully secure, low cost and based on readily available technologies. It is this system that we are using to compare remote BG monitoring with standard clinic care in the gestational diabetic population.

The evidence base for m-health as an intervention to improve glycaemic control in diabetes outside pregnancy is becoming increasingly well established.^9^ Our team members have had experience with developing and evaluating solutions for the management of type 1 and 2 diabetes using these approaches.^10–12^

There are few published studies of digital health solutions for the management of women with GDM. Most have enrolled only small numbers of women and have been conducted with methodological approaches at risk of bias. Comparison is hampered by the wide range of technologies that have been developed, with little agreement on the key components of interventions, the appropriate comparator group(s) or primary outcome measures.

Consequently, the true effects and possible benefits or harms of digital health solutions on GDM are not understood. While four studies of women with GDM (272 women) failed to demonstrate any impact on pregnancy outcomes or glycaemic control,^13–16^ other studies of women with type 1 diabetes^17–19^ or GDM^19^ showed an association with better glycaemic control and improved outcomes in the digital health group compared to standard care. It has been proposed that the value of telehealth solutions may be in reducing the number of outpatient clinic visits required,^16^ and there is evidence of improved satisfaction with care among patients and healthcare professionals.^13^ ^14^ ^19^ Sustainability and scale up of the technologies has proven challenging, with no examples in the published literature describing successful widespread implementation and adoption beyond the research setting of telehealth solutions for GDM.

## Methods and analysis

### Objectives

The primary objective of this trial is to evaluate the efficacy of monitoring BG in pregnancy using remote glucose monitoring (GDm-health) compared to standard clinic care. Efficacy will be determined by comparing BG control as determined by mean BG readings from recruitment until delivery between the intervention and the control group. The secondary objective will be to compare the two groups for compliance to the allocated BG monitoring regime, maternal and neonatal outcomes, glycaemic control using glycated haemoglobin (HbA1c) and other BG metrics, and patient attitudes to care assessed using a validated questionnaire^20^ and resource use.

### Study design

This is an open-label, individually randomised controlled trial of 200 women who have had an abnormal oral glucose tolerance test (OGTT) in their current pregnancy. Patients are randomised to the intervention (using the GDm-health management system) and half the number of clinic visits or to normal clinic care. Patient participation is from recruitment (a time point in pregnancy between 14 and 34 completed weeks of pregnancy) until delivery.

### The population studied

This trial is conducted in a large single centre tertiary referral unit in southern England. Pregnant women are screened for risk factors for GDM in their current pregnancy as per the National Institute of Care Excellence Guideline—Diabetes in Pregnancy.^5^ Women with previous GDM will be referred for a 2 h 75 g OGTT at 16 weeks and women with other risk factors for GDM are referred for an OGTT around 28 weeks of pregnancy. GDM is diagnosed by the International Association of Diabetes and Pregnancy Study Groups (IADPSG)^6^ criteria: fasting plasma BG ≥5.1 mmol/L; and/or 1 h ≥10.0 mmol/L and/or 2 h ≥8.5 mmol/L.

### Source and screening of potential participants

**Figure 1:**
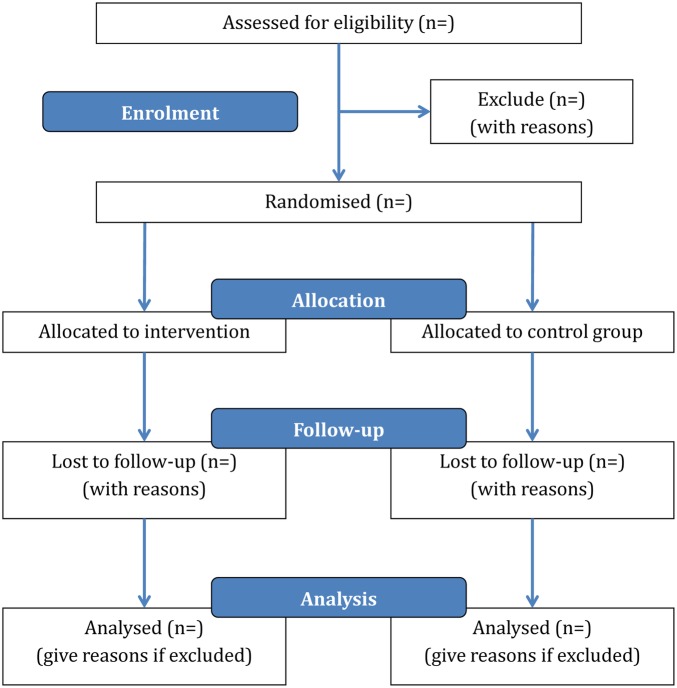
Flow diagram.

**Figure 2:**
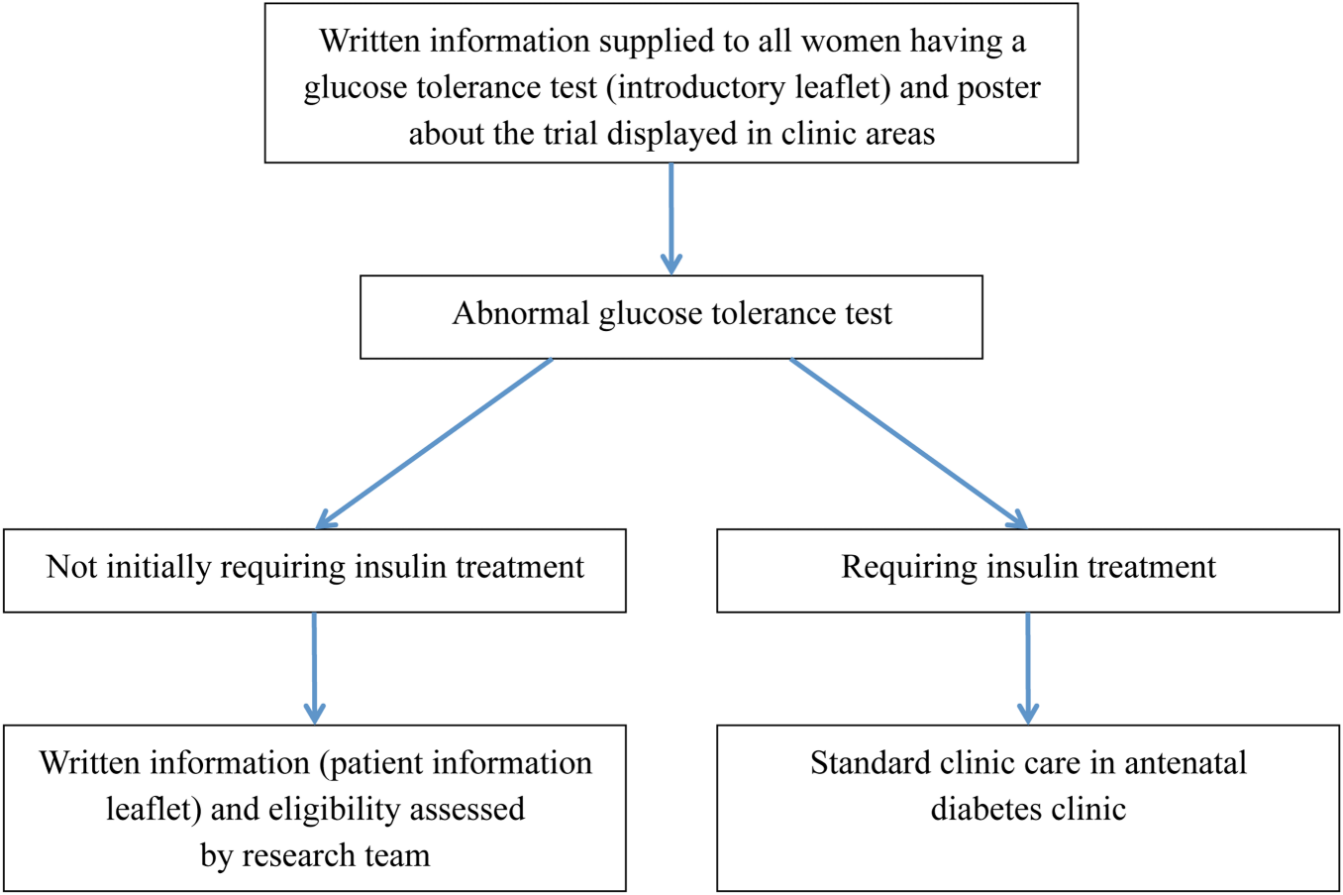
Route of initial approach for inclusion.

Pregnant women found to have GDM will be assessed by a specialist diabetes midwife. All women will be taught how to test and record their BG levels and asked to keep a food diary for 1 week. All women will be given an information leaflet about the study at their first visit. One week later, they will be assessed again in the specialist diabetes midwife clinic. At this visit, women will be given dietary advice and have baseline measurements taken (box 1). Those requiring insulin treatment for their gestational diabetes will start treatment and be referred to the consultant-led antenatal diabetes clinic. Those not requiring pharmacological treatment or only requiring oral hypoglycaemic therapy (metformin or glibenclamide) and that meet the inclusion/exclusion criteria listed in table 1 will be eligible for the trial and invited to participate. Figures 1 and 2 summarise the study selection process.

Box 1 **Measurements taken**

The baseline measures at recruitment:

1. Gestational age (days)
2. Weight (kg)
3. Age
4. Parity
5. Ethnicity
6. Smoking status
7. Highest education level
8. HbA1c (%)
9. Medication (type and dosage)
10. Blood pressure
11. Oral glucose tolerance test result

Measures taken at subsequent clinic visits

1. Glycated haemoglobin
2. Changes to medication
3. Weight (kg)

Maternal and fetal outcomes

1. Gestational age at delivery (days)
2. Birth weight (kg)
3. Mode of delivery (vaginal, caesarean, assisted)
4. Severe perineal injury (binary)
5. Shoulder dystocia/birth injury (binary)
6. Neonatal hypoglycaemia (binary)
7. Neonatal hyperbilirubinaemia (binary)

Diagnosis of maternal pregnancy-induced hypertension or pre-eclampsia (binary)

- Admission to higher level of care (maternal) (binary)
- Admission to higher level of care (neonate) (binary)

**Table 1:** Inclusion and exclusion criteria

Inclusion criteria	Exclusion criteria
An abnormal glucose tolerance test in this pregnancy (as defined by IADPSG recommendations^6^)	Impaired cognitive function such that she is unable to operate m-health equipment
Women not requiring pharmacological treatment at recruitment	Any evidence of fetal compromise
Women started on oral hypoglycaemic therapy at recruitment	Known risk factors for obstetric complications, other than obesity and gestational diabetes
Willing and able to give informed consent for participation in the study	Gestational diabetes requiring immediate insulin treatment
Female aged between 18 and 45 years	Twins or higher order pregnancy
Singleton pregnancy	Criteria for abnormal OGTT not met in this pregnancy
Able to travel to hospital independently	OGTT result suggesting pre-existing diabetes (ie, not gestational diabetes) defined as a fasting blood glucose of ≥7.0 or 2 h≥11.1 mmol/L^10^
	Gestation greater than 34+6 at the time of potential recruitment
	Unable to speak English well enough to explain or use equipment
	Used the GDm-health system in a previous pregnancy

IADPSG, International Association of Diabetes and Pregnancy Study Groups; OGTT, oral glucose tolerance test.

### Eligibility

The inclusion and exclusion criteria are listed in table 1.

### Consent

Written informed consent will be obtained by means of a dated signature from the woman and the person who obtained the informed consent. Consent will be obtained by the chief investigator or a qualified healthcare professional with delegated authority. A copy of the signed informed consent document will be given to the woman, a further copy will be retained in the woman's medical notes and the original retained by the chief investigator.

### Randomisation

Participants will be randomly allocated to one of two groups using a partial minimisation procedure. This will adjust the randomisation probabilities between groups to balance important covariates including gestational age at recruitment (<28 or ≥28 weeks), weight at randomisation (<90 or ≥90 kg) and ethnic group (Caucasian/white or other) using Oxford University Primary Care Clinical Trials Unit randomisation system—Sortition.^21^ This is an online randomisation programme with secure access for predesignated individuals (investigators from the trial). Randomisation will be carried out at the time of recruitment by the investigators by logging onto the system to obtain the next allocation. Women will be randomised individually into the study in an equal ratio of standard care (control) to remote monitoring (intervention). Both groups will use the same glucose meter (OneTouch UltraEasy, LifeScan, Inc).

### Treatment allocation

**Figure 3:**
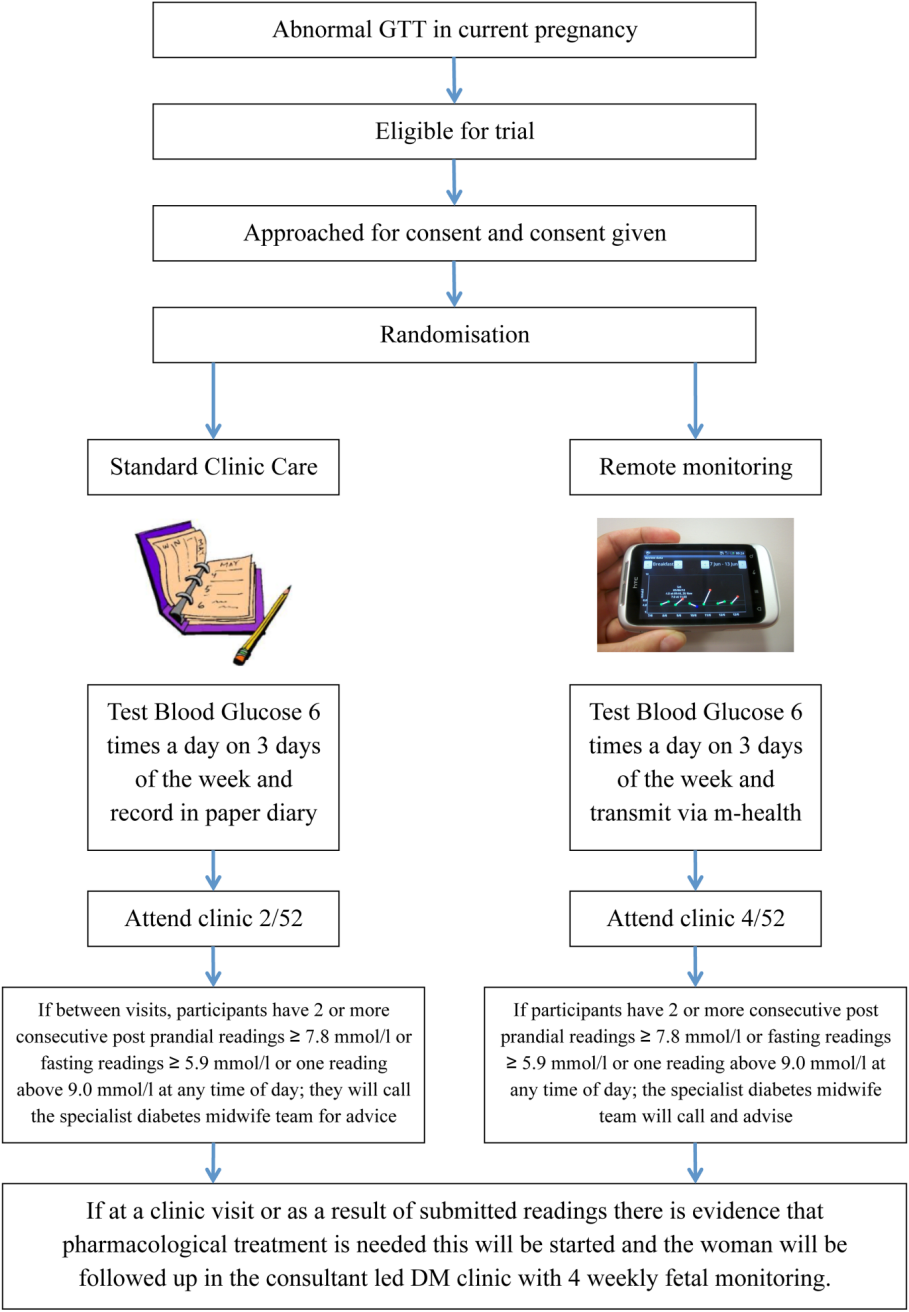
Study overview.

Women will be allocated to either the standard clinic care group (control) or the remote monitoring group (intervention) (figure 3).

BG monitoring targets, dietary advice and thresholds for starting medication will be the same for both allocation groups. All women will be asked to test their BG six times a day on at least 3 days of the week, as per the local guideline. This consists of a fasting sample, 1 h post breakfast, pre-lunch, 1 h post lunch, pre-dinner and 1 h post dinner. Optimal BG is a fasting reading ≥3.5 and ≤5.8 mmol/L and 1 h postprandial readings below 7.8 mmol/L.^11^ If there are persistently high BG readings, or medication is deemed to be required, women will be asked to increase monitoring to 7 days/week.

### Standard clinic care group

Participants in the standard clinic care group will self-record their BG values in a paper diary at home. They will be scheduled to attend the maternity diabetes clinic every 2–4 weeks where a midwife or specialist will review their readings. If, between visits, they have two or more consecutive postprandial readings ≥7.8 mmol/L or fasting readings ≥5.9 mmol/L or one reading above 9.0 mmol/L at any time of day, they will be instructed to leave a phone message for the specialist diabetes midwife. The midwife will return their call and provide further discussion and dietary advice within 72 h. If pharmacological treatment is required, the participants will be referred to the consultant-led diabetic clinic to start treatment.

### Remote glucose monitoring group

**Figure 4:**
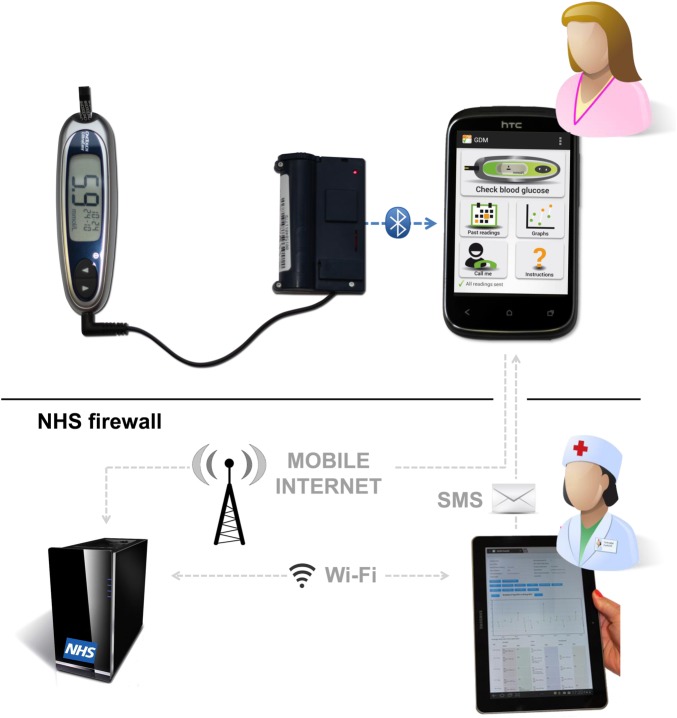
The GDm-health Management System.

Participants in the intervention group will be given a smartphone with a preinstalled GDm-health application and Bluetooth-enabled BG meter. The research team will teach intervention participants how to use the application (figure 4). Intervention participants will be asked to attend the clinic every 4–8 weeks (ie, half as many clinic visits as the standard care group). Healthcare professionals and participants will have access to different parts of the GDm-health management system. Participants will access the front-end, an Android smartphone application and healthcare professionals will access the back-end, a server hosted within the local Trust NHS network, through a web interface. The system is described in more detail below.

Participants will be invited to contact the specialist diabetic midwife using a call-back request function in the smartphone application, or by phone call, if they have any concerns. A specialist diabetes midwife will review the results of the remote monitoring at least three times a week. If a participant has two or more consecutive postprandial readings ≥7.8 mmol/L or fasting readings ≥5.9 mmol/L or one reading above 9.0 mmol/L at any time of day, the specialist midwife will call the participant or send an SMS message from the web interface to the participant’'s phone inviting discussion and further dietary advice. If fewer than 12 of the minimum 18 readings a week are being done, an automatic alert will be generated on the web interface for the diabetes specialist midwife. If this alert persists for 2 weeks, the specialist diabetes midwife will contact the participant. If there is evidence that pharmacological treatment is required, the participant will be referred to the consultant-led clinic to start treatment. Further dose adjustments and messages of advice/encouragement will be communicated to the participant between clinic visits via the GDm-health management system by the specialist diabetes midwife.

Measures taken in both groups at subsequent visits and after delivery are listed in box 1.

### Description of the GDm-health system

The development of the GDm-health system has been described previously.^8^

Each connection between the front-end (smartphone application) and the back-end (server and web interface) will be securely established on an encrypted channel and—for privacy reasons—the front-end will not contain any person-identifiable information (ie, participants will be identified by numeric ID). In order to avoid errors while reporting the BG measurement, the glucometer to be employed (LifeScan OneTouch UltraEasy) will be connected to the smartphone via a third-party Bluetooth module (Polytel Wireless GMA—Polymap). The LifeScan OneTouch UltraEasy is the glucometer of choice as it is compliant with the ISO 15197:2003 standard for blood glucose monitoring systems for self-testing in managing diabetes mellitus (this ISO standard was applicable at the time of the RCT). Readings will be transferred automatically to the Android application running on the smartphone at the time of the BG measurement, with no need of manual input.

As soon as the BG reading is stored in the smartphone's memory, the participant will be asked to ‘tag’ the reading as preprandial or postprandial through the smartphone application interface. Through the application, she will also be able to enter additional free-text comments as well as dosage and type of medication taken around the time of each reading.

All information will be transmitted to the back-end server automatically when the participant exits the application or as soon as the smartphone connects to the mobile network. The BG readings and associated information entered by the participant will be displayed together on the web interface for the benefit of the clinician reviewing the data.

Data review will be possible in two different ways: a table of all the readings taken by the patient listed in reverse chronological order and a graphical display of the readings to highlight the difference between preprandial and postprandial BG levels (values will be displayed as green, yellow or amber according to the corresponding alerting thresholds).

### Measures of BG

For the control group, BG data will be scanned at each clinic visit, with identifying labels removed, and stored in an anonymised file with each participant allocated a unique code. BG readings will then be transcribed from the paper charts, with BG results stored on a secure anonymised database. These data will be verified with readings downloaded from the patient's glucose meter at the end of the pregnancy using the same unique ID for later comparison. The intervention group data will be extracted from the website and stored on a secure anonymised database using a unique ID.

### Accrual and analysis

#### Sample size

The four published randomised trials that investigated the effect of a telehealth intervention on mean BG control in pregnancy reported SDs between 0.2 and 1.2 mmol/L. The lower value was from a very small study (n=19) conducted in 1996.^22^ The range of SD for mean BG in the three relatively large studies (n>30) was 0.5–1.2 mmol/L.^13^ ^15^ ^17^ This indicates that we do not yet have a precise estimate of the likely SD in mean BG, making sample size calculation challenging. We therefore pragmatically decided to assume an SD of 0.8 mmol/L for the mean BG level at the end point. Thus, with 100 patients in each arm, we will be able to detect a difference between the arms of 0.32 mmol/L, with power of 80% and a significance level of 0.05.

The multilevel analysis we propose has not been conducted in any of the previous studies and will use all the data from recruitment to delivery. Thus, the difference in BG between the arms at the study end point will be estimated more precisely compared with a trial that uses a comparison of mean BG at the end point alone or a mean of all readings. Therefore, the calculation given provides a conservative estimate of the effect size that could be detected.

### Statistical analysis

#### Descriptive analyses

Baseline demographic characteristics of each group will be tabulated. The number of patients, number of missing values, the mean, range and SD of continuous variables, and the frequency of the binary variables will be reported.

The analyses will be based on the intention-to-treat (ITT) population, which will include all patients randomised. A per-protocol analysis will also be performed excluding women who did not use the GDm-health system for at least 67% of expected BG readings (at least 18 readings per week) to determine the efficacy of the system under optimal use. This level was selected as it was programmed at the beginning of the trial as the level at which the computerised system would alert healthcare professionals that a woman was non-compliant with system use.

### Analysis of end points

#### Primary end point analyses

The primary end points will be efficacy of the GDm-health system to achieve glycaemic control compared between groups. The results will be reported as a comparison between the two groups of change over time of BG level. The complete data form a complex longitudinal data set, over gestational age. A mixed-model analysis will be carried out on the data of the glucose level. The BG is measured six times per day according to the prescribed schedule, and levels of the multilevel analysis will be time point, gestational age and patient. In addition to the repeated measurements of BG, patient covariates (age, body mass index (BMI), gestation at recruitment, ethnic group) will be included in the model as fixed effects.

#### Secondary end point analysis

*Markers of BG control and management intensity*

- *Analysis of the HbA1c data:* These data will form a longitudinal data set, with the measurement of time being gestational age. The number of assessments per patient will vary. Some patients may be lost to follow-up. The correlation between repeated measurements on each patient and the variable number of measurements will be taken into account in the mixed-model analysis. In addition to the repeated measurements of HbA1c, patient covariates—age, haemoglobin at recruitment, BMI, gestation at recruitment and ethnic group—will be included in the model as fixed effects. The results will be reported as a comparison between the two groups of the rate of change of HbA1c with time.
- *BG analysis:* Other exploratory BG metrics will include:
  Overall mean BG for fasting, preprandial and postprandial readings;
  Percentage of readings ‘on target’ (fasting readings as defined ≥3.5 and ≤5.8 mmol/L and postprandial readings ≥3.5 and ≤7.7 mmol/L) for the first 4 weeks after randomisation and the second 4 weeks after randomisation;
  The value, and week number after randomisation, of the highest weekly mean BG
  Time to treatment from recruitment in weeks;
  Number of dose adjustments of hypoglycaemic drugs;
  Maximum dose of insulin and/or oral hypoglycaemic therapy (metformin).

*Maternal and fetal outcomes as measured by:*

- Gestational age at delivery (and percentage of deliveries before 37 weeks);
- Birth weight (and percentage of babies with birth weight above 90th centile as per the INTERGROWTH-21st Standards for gestational age and gender);
- Mode of delivery (vaginal, caesarean, assisted);
- Perineal trauma (third-degree or fourth-degree perineal tear or tear requiring suturing in the operating room);
- Incidence of shoulder dystocia/birth injury;
- Incidence of neonatal hypoglycaemia (defined as a serum BG level of <1.5 mmol/L at any time, or requiring special care baby unit (SCBU) admission for feeding);
- Incidence of neonatal significant hyperbilirubinaemia (defined as requiring phototherapy indicated by a serum bilirubin level of >200 μmol/L at <35 weeks, >250 μmol/L at 35–36 weeks and >300 μmol/L at ≥37 weeks);
- Maternal weight gain (adjusted for weeks in trial);
- Maternal pregnancy-induced hypertension or pre-eclampsia (defined according to the International Society for the Study of Hypertension in Pregnancy (ISSHP) criteria^23^);
- Admission to higher level of care for mother;
- Admission to higher level of care for neonate.

*Maternal and neonatal outcome analysis*: An analysis of covariance will be carried out when the outcome variable is continuous. The model will contain the term for randomisation group and baseline covariates. The results of a comparison between the two groups will be reported as a treatment effect with 95% CI. The binary variables will be analysed using logistic regression. The model will contain a term for randomisation group and baseline covariates. The results of a comparison between the two groups will be reported as an OR and a risk difference with 95% CI.

*Participants' attitudes*

- Patients' attitudes will be assessed by completion of the Oxford Maternity Diabetes Satisfaction survey. This survey was developed based on several existing satisfaction surveys for diabetes management and was adapted and validated within this population.^20^ Descriptive analysis of the scores will be presented and thematic analysis of free-text comments performed.

*Compliance with the BG monitoring system*: Summary statistics will be calculated to describe the frequency of BG measurements.

An exploratory economic investigation of the TREAT-GDM data will take the form of a detailed cost analysis from an NHS and personal perspective of the healthcare treatment pathways of pregnant women using the digital health intervention and those receiving standard clinic care. For the NHS perspective, the exploratory analysis will aim to identify cost differences between both groups in terms of healthcare resource use during delivery, neonatal care, antenatal clinics, redistribution of time for health professionals and medication and associated titration. Personal care categories will concentrate on estimating travel-related costs. A thorough cost analysis of adopting the digital health intervention at a hospital level will be reported. We will report unit costs, resource use and costs separately between treatment arms as restarted by best practice guidelines.^24^

*Economic analysis*: The statistical analysis used for the exploratory economic analysis will be primarily descriptive, and parametric inferences to detect potential statistical cost differences between arms will be conducted only on identified potential cost drivers in the exploratory analysis. For the latter, mean cost differences will be accompanied by appropriate measures of uncertainty including 95% CIs.

Interim analyses will not be performed, and all analyses will be performed after the end of the study. Analyses will be blinded to allocation group.

### Safety

Supervision in the form of weekly meetings with the clinicians, research team and the diabetes midwives will occur to ensure adherence to the protocol and to resolve problems and queries.

The use of self-capillary BG monitoring is well established in the pregnant and non-pregnant population. Safety concerns include adequate alerts if the remote glucose testing groups have either high or low BG readings or are not providing readings. This will be addressed by having automatic alerts activated to ensure abnormal results are not ‘missed’. The equipment to be used is a standardised, approved glucose meter with Bluetooth technology. It has been used in numerous previous studies and found to be robust. Decisions about patient care will continue to be made by trained doctors and midwives. The technology will not be relied on to automatically alert patients or provide automatic changes in management in response to the readings the system receives.

The m-health system uses a standard BG meter (OneTouch UltraEasy, LifeScan, Inc), which connects to a smartphone (HTC Desire C with Android Operating System V.4.1) via a commercially available Bluetooth adapter (Polytel Wireless Glucose Meter Accessory (GMA), Polymap Wireless LLC). The users measure their BG in the same way as usual care, but the GDm-health system allows a copy of the readings to be transmitted to the smartphone via Bluetooth. The readings are then transmitted to a secure NHS server in real time via the mobile phone data network, using checksums and encryption to ensure data integrity. The BG readings are stored in the user's meter, as well as in the smartphone memory and on the NHS server. No patient-identifiable data are transmitted between the meter and the phone or between the phone and the NHS server.

A business continuity plan has been developed to ensure women using the GDm-health system and their healthcare professionals have a contingency plan if the system fails.

## Ethics and dissemination

### Data handling and record-keeping

All study data will be entered on a password-protected, electronic spreadsheet within the University of Oxford high-compliance secure drive. Participants will be identified by a study-specific ID number. The key to these study numbers will be maintained on a single document in a locked cabinet in an office to be accessed only by authorised personnel via a code. The names and any other identifying details will not be included in any study electronic files. All data will be double entered by two researchers. Clinical and laboratory data will be extracted from the patient notes and the Oxford University Hospitals NHS Trust electronic patient record system. Data from all participants, including those who drop out of the study, will be kept for 5 years, as per university policy. Hard copies will be kept in a locked room and electronic copies will be kept in a password-protected file.

### Ethical issues

The same treatment algorithms will be used between the two groups; therefore, the clinical care received will strive to be similar, although the method of delivery of that care will differ. To ensure hardware and patient satisfaction questionnaires are returned, each participant will receive a £20 shopping voucher once they have sent back their meters, phones and questionnaires.

### Publication policy

The chief investigator will co-ordinate dissemination of data from this study. All publications using data from this study to undertake original analyses will be submitted to the Clinical Investigators Group (CIG) for review before release. To safeguard the scientific integrity of the trial, data from this study will not be presented in public before the main results are published without the prior consent of the CIG. We will submit the results of this study for peer-reviewed scientific journals and conference presentation. All authors will have publication rights. This trial is registered at clinicaltrials.gov NCT01916694.

## Discussion

This trial will give valuable information regarding the role of remote BG monitoring in women with GDM. We will determine the efficacy of remote monitoring in pregnant women with GDM compared to conventional care and provide an objective assessment of the potential value of the technology in impacting outcomes including BG control, patient satisfaction and resource use. Severe adverse clinical outcomes, such as shoulder dystocia or neonatal hypoglycaemia, associated with GDM are relatively uncommon among women receiving management. Therefore, we will use a measure of glycaemic control as our primary outcome as this has been shown in large studies^3^ ^4^ to correlate with risk of adverse outcomes.

Selecting a clinically relevant summary measure of glycaemic control in pregnancy is challenging owing to the progressively changing physiology of glucose regulation in the third trimester of pregnancy. We plan to assess the difference in overall mean BG between the two groups as the primary outcome for this trial; however, an intended objective will also be to assess and compare other summative metrics of glucose control including HbA1c, changes in BG over gestation and changes in BG in relation to meal times between the two groups. This will enable us to select a valid primary outcome measure, with known SD and estimation of effect size, to allow calculation of sample size for a future definitive trial.

One of the criticisms of the vast number of health-related ‘apps' now available is that there is currently little empirical evidence to support their use. Most ‘apps' have been developed by the commercial sector or by patient groups, but few are integrated within the healthcare system. We hope that the results of our trial will provide some of the first robust findings on the potential for integrated remote monitoring systems to affect patient outcomes.
